# “*Who'll do all these if I'm not around?”*: Bonding social capital and health and well-being of inpatients

**DOI:** 10.1080/17482631.2018.1435108

**Published:** 2018-02-15

**Authors:** Padmore Adusei Amoah, Adwoa Owusuaa Koduah, Razak Mohammed Gyasi

**Affiliations:** ^a^ Division of Graduate Studies and Asia Pacific Institute of Aging Studies, Lingnan University, Tuen Mun, Hong Kong (SAR); ^b^ School of Nursing, The Hong Kong Polytechnic University, Hung Hom, Kowloon; ^c^ Department of Sociology and Social Policy, Lingnan University, Tuen Mun, Hong Kong (SAR)

**Keywords:** bonding social capital, healthcare delivery, inpatients, health and well-being, Ghana

## Abstract

**Purpose**: Although social capital influences health-related decisions and behavioural patterns in many developing countries, minimal attention has been paid to the nuances of its effect on healthcare. This paper examines how bonding social capital affects healthcare delivery for inpatients in Ghana.

**Methods**: Semi-structured in-depth interviews were used and thematic analysis method employed to analyse the data. Interviews were conducted with health professionals and relatives and close friends of inpatients in three public health facilities in Ashanti region.

**Results**: Relatives and close friends of inpatients were a critical source of instrumental support such as provision of meals, laundry services, running errands and financial assistance as well as emotional support. These functions—that were both ‘expected’ and ‘encouraged’— reduced the burden on the health facilities, which apparently had limited resources to offer adequate care. However, the relatives of inpatients sometimes inadvertently obstructed efficient healthcare delivery through actions such as extending ‘unapproved’ alternative care to patients. Moreover, the process of contributing towards health and well-being of the sick exposed the relatives to health risks due to poor living conditions.

**Conclusion**: A well-defined and befitting role must be devised for at least an immediate social relation of inpatients to improve the positive effects of bonding social capital on healthcare delivery.

## Background

Social capital is a key determinant of health and health-related behaviour and choices in many contexts and among different population groups (Kawachi, Subramanian, & Kim, 2008; Yip et al., 2007). It refers to the actual and potential resources embedded in different forms of social relationships (Bourdieu, 1986). The resources embedded in social relationships are often in the form of information, instrumental help (e.g., money and running errands) and emotional support (Harpham, 2008; Harpham, Grant, & Thomas, 2002). The reliance on social connections for health-related assistance is said to be more pronounced among relatively vulnerable persons (Amoah & Jørgensen, 2014; Avogo, 2013; Narayan, 1999; Yip et al., 2007). In Ghana and many other developing countries, not only have fewer studies been carried out about social capital (Amoah, 2017; Story, 2013), but also minimal attention has been paid to the role of the phenomenon in actual healthcare delivery. Moreover, considering that developing nations do have weak and complicated health systems (Barnes, Brown, & Harman, 2015; Smith & Hanson, 2012), social capital can offer precise perspectives and possibly, solutions to some of the recurrent challenges in healthcare delivery (Hollard & Sene, 2016). Such challenges, among others, include cultural incompetence of many health systems. The World Health Organization (WHO) advocates for intercultural healthcare strategies (Gyasi et al., 2017; WHO, 2013). Given this, the study of the role of social capital in healthcare delivery is even more critical for weak health systems such as that of Ghana (Saleh, 2013). Many cultural elements and practices are transmitted through both conscious and unconscious social interactions (Halpern, 2005).

Based on the premise that health systems are embedded inherently in social relationships (Gilson, 2003), this paper examines the role of, notably Bonding Social Capital (BSC)—in healthcare delivery for inpatients. For purposes of this study, inpatients consist of persons admitted to general hospital wards for various physical health problems. BSC refers to tight-knit groups or networks that are similar regarding demographic factors, such as age, ethnicity and education (Halpern, 2005). A typical example is a bond between family members and close friends. BSC is different from other forms such as bridging, and linking social capital as it is the commonest and perhaps most accessible form of social capital (Halpern, 2005; Szreter & Woolcock, 2004). On the one hand, bridging social capital constitutes relatively weaker social ties that cut across identities (e.g., race, ethnicity, religious affiliation), and social class and tend to have a broader reciprocity (Musalia, 2016; Szreter & Woolcock, 2004). On the other hand, linking social capital represents a hierarchical relationship or unequal relationship between individuals with differences in power, resources and social status. It can also refer to relationships between people and institutions (Amoah & Phillips, 2017). Bonding, bridging and linking social capital are part of what is termed as structural social capital, which is different from cognitive social capital (Halpern, 2005; Musalia, 2016; Szreter & Woolcock, 2004). Cognitive social capital manifests in the form of abstract elements such as trust, sense of fairness and harmony (Amoah & Phillips, 2017; Musalia, 2016). The paper also attempts to clarify the dilemma about the role of resources embedded in tight-knit associations—like the relationship between informal care and utilization of formal healthcare—either as a substitute or complement to [formal] healthcare delivery for people on clinical admission (Reeves et al., 2014). Indeed, very little attention has been given to mobilizing individuals and communities towards strengthening healthcare delivery according to the Ministry of Health of Ghana (MoH, 2007).

In clinical settings, the necessity of BSC is recognized mostly by the palliative care philosophy which acknowledges the close ties of patients as essential “unit of care” (Keating & Dosman, 2009). Considering that pressures on health facilities and personnel have resulted in limited clinical encounters and minimal personal relationships between patients and health personnel, the role of tight-knit networks of patients have become even more pertinent (Gage, 2013). Availability of BSC may help in effective information transmission to patients (Labrecque, Blanchard, Ruckdeschel, & Blanchard, 1991; Mayberry, Rothman, & Osborn, 2014). Labrecque et al. (1991) attest from earlier clinical observations that physicians spend more time and transmit more information to patients whenever family members are present during the consultation (Labrecque et al., 1991). However, the presence of close social ties also tends to obstruct communication between health personnel and patients through “nagging” and misapplication of vital health information support (Mayberry et al., 2014). The work of Mazer, Cameron, DeLuca, Mohile, and Epstein (2014) who opine through empirical clinical studies that families and relatives of sick persons sometimes act as pseudo-surrogates by conveying health information from health professionals to sick persons even when the sick is present and capable of handling the interactions collaborates this assertion. These situations, according to the researchers, potentially create distortions in transmission and application of vital health information.

However, the presence of close social acquaintances ensures continuous care for especially people with chronic ailments (Roddis, Holloway, Bond, & Galvin, 2016). A study among recurrent inpatients showed that construction of strong social ties was crucial to reducing hospitalization (Andreasen, Lund, Aadahl, & Sørensen, 2015). In many sub-Saharan African countries, the influence of relatives and sometimes neighbours, on access to and use of health services work, partly because of “reverse responsibility”. For instance, in a study to gain an understanding of the unusually high adherence to antiretroviral therapy for HIV/AIDS in three sub-Saharan African countries, it was found that the sick persons deliberately sought and received support to access such services. However, the helpers ensured value for money by making sure that the patients adhered to the therapy (Ware et al., 2009). BSC, therefore, serves not only as a source of a resource such as financial support as has been witnessed in many contexts but also provides a buffer against poor attitudes towards needed care (Ware et al., 2009). Moreover, participation in care delivery by close ties of inpatients assists in adopting values and concerns of the patients by health professionals (Mazer et al., 2014). In Ghana, such values often include religious and other belief systems, which shape everyday choices about health (Powell-Jackson & Ansah, 2015). However, while being sick ensures increased care and support from close social ties, such conditions are also known to lead to social network attrition through the loss of critical support that is not offered as part of clinical treatments (Halpern, 2005; Lewis, DiGiacomo, Currow, & Davidson, 2014). For instance, a study among frequently hospitalized persons in Denmark’s health sector observed that chronic illness reduces contact with close social ties and subsequently weaken said relationships (Andreasen et al., 2015). Furthermore, like other forms of social capital, the effectiveness of BSC is predicated on cognitive social capital elements such as trust, norms, identities and harmony. These elements are adjudged as the glue that holds social relationships together (Edwards & Foley, 1998; Van Vugt & Hart, 2004). Indeed, people are more likely to offer social and professional support for health purposes in situations where cognitive social capital is present (De Silva & Harpham, 2007; Gilson, Palmer, & Schneider, 2005; Østergaard, 2015). This study evokes how these theoretical and empirical observations play out among inpatients in general wards in Ghana.

## Methods

### Study design and context

This work is part of an ongoing larger study, which examines the tripartite relationship between social capital, healthcare access and health literacy among rural and urban adult populations in Ashanti Region, Ghana. The data for the original study were gathered from June to October 2015 drawing on a cross-sectional design. This paper emanates from the qualitative data and findings of the main study, which sought to explicate the complex mechanism of social capital’s effect on health and well-being among the public and the sick. This was in an attempt to reinvigorate the role of, and how social elements can be integrated into healthcare delivery. The paper leans towards the interpretivist and subjectivist epistemology—the idea, that what we can know of reality is socially constructed through our intersubjective experiences within the lived world (Angen, 2000; Bryman, 2012). Thus, the study emphasized the participants’ perspective of the subject matter. The biases and limitations of this philosophy (Angen, 2000; Bryman, 2012), were however reckoned. The research process employed a thorough validation process to assure trustworthiness of the study. First, an ethical validation was carried out. Largely, this involved adherence to the admonition to “do everything we can to see to it that the debate is fair, that no one’s [participant’s] voice is excluded or demeaned” (Caputo, 1987, p. 260). Further to that, the process involved a commitment to self-reflexivity through continuous consideration of our understanding—including political and theoretical prejudices—and that of others of the subject matter (Angen, 2000).

### Participants and data collection

The study mainly used semi-structured in-depth interview guide (Bryman, 2008) to elicit data from 14 primary participants and five key informants—health personnel or medical officers. These data were gathered at one tertiary hospital (Okomfo Anokye Teaching Hospital) and two secondary health facilities (Ejisu-Juaben District Hospital and Old Tafo Government Hospital). The three health facilities presented different categories of primary participants regarding their origin (both rural and urban users) and the kind of support they offered due to differences in functions. Moreover, these facilities have strategic locations within the Metropolitan area with regard to physical accessibility to people even in the entire region. Purposive and snowball sampling techniques (Bryman, 2012) were applied to select the primary participants. The inclusion criteria for the primary participants consisted of adults (18 or above) whose relatives were on admission in the three facilities for at least three days. The protocol did not specify persons with relatives who had particular ailments. However, the focus was on those with relatives in general wards. Thus, relatives of patients admitted for nonintensive care for both communicable and noncommunicable diseases. This group was chosen to capture the diverse role of BSC, as patients in general wards were more accessible to relatives. The criterion of admission duration was in consonance with the average length of stay (ALOS) at hospitals in Ghana (Saleh, 2013). The participants were identified from the relatives of inpatients who waited in and around the respective health facilities aimed at visiting the sick or participating in the healing process. Indeed, it is quite common to find such persons in and around hospitals in the study region and many other parts of Ghana (Baneseh, 2016). Participants who were identified earlier were asked to assist in locating others who met the inclusion criteria.

Six of the primary participants were from Old Tafo Government Hospital, five from Komfo Anokye Teaching Hospital and three from Ejisu-Juaben District Hospital. The majority of relatives and friends of inpatients found in and around the hospitals’ premise were females. Consequently, the participants were mostly females (71%). Most of them were married (64%) and had an average age of 44 years. Junior High School was the commonest highest educational attainment amongst them. Some of the participants had travelled long distances to be with their friends and relatives on admission. One of the participants (female, 42 years) was from the Western Region of Ghana. All the others were from the study region although from different localities and districts.

The health personnel were included to provide professional/clinical perspective to the experiences and testaments of the primary participants. The health personnel consisted of two physicians (general practitioners) and three general nurses. These two categories of health personnel were chosen because they are often the closest to patients and their relatives regarding interactions during care. One nurse was selected from each of the hospitals. The physicians worked at Old Tafo Hospital and Ejisu-Juaben District Hospital. For both participating groups, students (nurses and physicians) and those in temporary positions were not included in the study. Aside from that, each of the health personnel must have worked at least one year in their current health facility. It was anticipated that one might have gained enough contextual and relevant experience after a year in service in a particular health facility.

The in-depth interviews captured how the relatives of patients on admission contributed and affected the process of healthcare delivery from the perspective of the relatives and health personnel to provide a balanced response to the research aim. The items for the interview were adapted from the “individual support part” of the short version of the Adapted Social Capital Assessment (S-ASCAT) instrument (Harpham, 2008; Harpham et al., 2002). Participants were asked whether they had received support—whether informational, emotional or instrumental forms—from different categories of social relations in the past year in the original instrument. In the absence of the sick persons themselves, participants in this study were asked to elaborate how they contributed to the healing of the sick; and what engendered them to stick around the hospitals and how they oscillated between their views and experience and expert (health personnel) advice. The health personnel were asked to share their experiences in dealing with the family and friends of inpatients and how such acquaintances improved or impeded efficient care delivery. Elaborative questions such as “can you explain that further?” was used to draw in-depth knowledge from participants. All the interviews were conducted using the local language—“Twi”—except for two of the key informants who were interviewed using English as they could not fluently express themselves in the native language. The other three health personnel moreover drifted between English and “Twi”. The native language was used for interviews with primary participants due to linguistic limitations as regards English, which was imputable to the low educational attainment.

The health personnel were interviewed in their offices whereas the primary participant interviews were carried out in locations jointly selected by the interviewer and participants usually at a quiet spot within and around the health facilities. The interviews lasted an average of 45 minutes. Each session was tape-recorded with expressed consent from the interviewees.

### Ethical approval

The Committee on Human Research Publication and Ethics (CHRPE) of School of Medical Sciences, Kwame Nkrumah University of Science and Technology and Okomfo Anokye Teaching Hospital, Kumasi, Ghana, (CHRPE/AP/345/15) approved the study. Moreover, a written permission was received from the Ashanti Regional Health Directorate under whose jurisdiction the selected health facilities operated. Verbal consent was obtained from all participants after explaining the purpose and content of the study to them. Furthermore, pseudonyms have been used for each participant in the paper to ensure confidentiality and anonymity.

### Data analysis

The interviews were transcribed verbatim within 48 hours after completion. One local language expert validated the transcripts of interviews that were carried out using the native language independently. The audio-recordings were replayed and compared with transcripts prior and during the analysis. The analysis was primarily guided by a posteriori inductive approach—by inferring the implications of the findings for the theory of BSC (Angen, 2000; Bryman, 2012). Participants’ innate beliefs and everyday practices informed the analyses in keeping with the interpretivist position of the paper (Angen, 2000; Bryman, 2012). Essentially, this approach prioritized the recurrent concepts and messages per the accounts of the participants using thematic analysis technique (Bryman, 2012). The process entailed “open coding” (Bryman, 2012), to identify initial concepts which were later recategorized into themes—based on similarities and contrasts. The initial themes centred on the contributions and effects of close-ties of inpatients and the mechanism of influence. The themes were later juxtaposed. In doing so, some of them were merged while others were removed based on their significance to the study. The new themes were presented as findings primarily through narration and interpretative reasoning to ensure continuous linkage among the codes and sub-themes (Bryman, 2012). Finally, one social health researcher and one health personnel verified the themes and the respective codes independently.

## Findings

The findings present the resources embedded in the tight-knit social networks of sick persons on admission in health institutions as a key contributor towards healing through instrumental and emotional support offered to the inpatients and health personnel. However, BSC showed negative effects on operations of the health facilities through obstructive behaviour of the relatives. Furthermore, relatives of inpatients had to endure unsavoury conditions while they attempted to offer support. These observations are expatiated below:

### An expected social practice for healing

In the course of healthcare delivery, it was a common practice for close ties of the sick to contribute towards healing. Some relatives, therefore, expressed a sense of “responsibility” in response to the social expectations of availing oneself to the needs of the sick as one participant shared:

*I need to be around for anything the doctors want from my sister [the patient]. …She is very weak from her ailment. …I provide warm water for a bath every morning and evening*. (Eno, female, 40 years)

From a clinical perspective, the presence of social acquaintances exudes emotional support, which aids in healing and necessary for the well-being of even the terminally sick as some health personnel alleged. Perhaps, this is why the participation of relatives and other social acquaintances of the sick was both expected and encouraged to ensure quick recovery as one health personnel stated:

*Relatives are supportive. …For some people, the only time they receive affection from close relatives is when they are ill. They get anything they ask from their families. …There have been instances where patients on admission would whisper to me not to be discharged yet so they can continually enjoy the care and attention their relatives provide. …Because when the person is at home, nobody cares. …If the person is in the hospital, he gets the best meal, gets money, all the relatives are around him/her, and they feel happy which helps the patient recover quickly. …Hardly have I seen a patient on admission without the relatives or even friends coming to give one kind of support or the other* (Health Personnel #4)

Indeed, the views expressed by both participant groups portrayed the role played by close social ties as a mere fulfilment of expected social functions.

### A major source of financial support

A common form of support and taking a cue from the earlier observation, responsibility, was in the form of financial support. Indeed, financial support emerged as a primary responsibility of the close acquaintances of the sick as the health personnel placed the financial well-being in their hands and even the present extended social networks of the sick. Financial support did not emanate only from family members. Close friends, as some participants described themselves, were also critical for health and well-being of inpatients. This was particularly true for people in single households:

*I am his [the patient] friend. He is suffering from Malaria. He was admitted last Thursday, so he has been here for five days. He lives alone and has no one else to support him so I have asked my wife to cook for him every day while he is here. I also bring him money for upkeep and pay some of his bills* (Abro, Male, 41 years).

*Recently, a man was rushed here* [hospital] *by his colleagues at work after a heavy metal block fell on his foot. He was almost unconscious, as he had lost a lot of blood. …His friends contributed to making part payment of his treatment cost….After the second day, we needed more money for his treatment, he* [the patient] *asked us to call his friend for help. …The friend footed all the bills and items for his rehabilitation including clutches* (Health Personnel #3).

However, while the evidence affirms the financial support through BSC for healthcare purposes, not everyone was ready to tap into such resources. This was manifested by the unwillingness of some participants to seek medical attention for fear of being admitted owing to financial challenges. Hesitation in activating such resources stemmed primarily from social expectations such as the need to be financially independent as some participants hinted:

*It does not usually happen to me but when I am sick, and there is not much money for healthcare I do not let other people see it. It is difficult for me to disclose my situation to others. …People have money but if you ask for help, they will not only deny you the assistance but will also make you a laughing stock afterwards* (Paulina, 20 years, Female).

Evidently, there was some social stigma attached to the habit of relying consistently on one’s closed networks for assistance even if the resource holders were willing to part with them.

### Provision of ancillary services

From the perspective of the relatives of the sick, they were duty bound to ensure the well-being of the sick. Based on previous experiences, many of the participants demonstrated in-depth knowledge of the nature of and kinds of services offered by the respective health facilities. With the deficiencies of the facilities in mind, some participants attested to providing meals and laundry services in support of their relatives:

*“…I come here every morning and evening to check on my niece who is on admission here (Hospital). …In the morning, I bring her breakfast and take her dirty clothes home for cleaning. Later in the day, I bring her dinner and clean clothes. …The nurses have shown me how to prepare her food and the type of foods she needs* (Akoma, 44 years, female).

In the opinion of the caregivers and even health personnel, the ancillary services offered by relatives of the sick were crucial not only to the well-being of sick but also to the effective functioning of the hospitals. Services such as running errands—for instance in getting laboratory tests done in some situations—were of the critical operational essence:

*“I have been here for close to two weeks now. …It is my son who is sick. …We live far from here, and I do not have money to shuttle here every day. …Besides, I need to be around to help the doctors. Sometimes they ask for laboratory tests and sanitary items.I have to be here to provide them. …I take him to the laboratory for tests and get the results to the doctors when it is ready. …Who'll do all these if I not around?* (Nsiah, 51 years, male).

Indeed, it was the participants’ awareness of the deficiencies in the health delivery system that triggered their insistence on being close to the facilities which hosted their relatives to provide services that may be required but not offered. Considering their knowledge of the system’s deficiencies, some participants, unsurprisingly, held the opinion that their mere presence was enough to draw the rapt attention of the health professionals towards their relatives. Familial presence was regarded as a caution to health officers to act professionally and perform creditably:

*‘I am speaking from experience, my brother. …I do not spend the night here, but I come here every morning and evening. I make sure the nurse or doctors see me and other visitors around my mother’s bed [the sick]. …Once they [health personnel] know that the relatives of a patient are around and provide whatever they ask, they are more careful with your patient. …They make sure they do not make mistakes* (Amin, 43 years, Male).

This position was based mainly on hearsays and previous experiences of poor handling and condescending attitudes of health personnel towards patients, which had led to piteous health outcomes. The health personnel however disputed such claims:

*They think that their presence will make us do more for their relatives but that is never the case. We do our job without any favour or fear. ...As I said already, even their mere presence help in healing aside from the other services such as meals they provide* (Health personnel #4).

Thus, regardless of the positional differences, there appeared to be little doubt as regards the all-important role of BSC in healing and well-being of the sick through the provision of ancillary support.

### Obstructive and nuisance behaviours

While the health professionals advocated for relatives to visit and spend time with the sick due to its benefit to healing, some relatives behaved in a manner that inhibited efficient care delivery. The mere presence of social relations and their constant inquiry about the health status of the sick were sometimes a nuisance as some health personnel complained:

*The community members feel that they own the hospital so sometimes they behave in an affront manner. …Our rule is that by 8:00 am every relative is supposed to leave the ward to allow the nurses to clean-up and prepare the patients for ward rounds. …However, I got to the main ward at 9:00 am this morning, and it was full of non-patients. …Some were even physically attacking the nurses. I had to drive all of them out forcefully. …I think they do that because of inadequate knowledge of the operations of the health facility. They could even carry pathogens home if they spend long periods in the ward* (Health Personnel #2).

This attitude of relatives, however, was tied to the meaning they ascribed to the treatment regimen. Some of them took the health and well-being of the sick into their own hands by extending informal and alternative treatment methods to the sick at the blind side of health personnel:

*Some relatives would come to the wards during visiting hours to smear* [holy] *oil (from their religious leaders and spiritualists) on the sick to aid in recovery. …Some of the patients discuss new symptoms of their ailments with their relatives who in turn take matters into their hands instead of relaying the information to us* (Health Personnel #5).

Such incidents, in part, portrayed an ongoing conflict between different healing modalities in the study context. From the perspective of orthodox medical professionals, the behaviours of the relatives posed fundamental challenges to their practice. The health personnel found such intrusions—regardless of the intent—inappropriate. In fact, some relatives with decision-making powers sometimes held varying views and positions, which obstructed the flow of healing care. For example, there were instances where families would apply to withdraw their sick ones out of the hospital in the course of treatment on religious grounds:

*Occasionally, we encounter situations of patients with HIV or diabetes whose relatives will refuse treatment. …The family members will come to the doctor in the course of the treatment to request for release of their relative….. They would say ‘our pastor has informed us that the condition you are treating is not supposed to be treated at the hospital [orthodox medical practice], so we want to take him/her to the church’. …If the family still insists on the release of the sick after we try to explain the treatment plan to them, we ask their leader to write a letter for discharge against medical advice* (Health Personnel #1).

Despite the doubts some of them cast about the reasons given by the relatives, the health personnel could not independently validate such decisions. The doubts often aroused from bearing witness to the struggles of some families concerning funding. Mostly, such decisions by the relatives were colossal interference in the process of healing in the opinion of health professionals although due respect was always—as many claimed—granted to the wishes of the relatives.

### Caregivers becomes “squatters”

Although many social relations aimed at assisting their sick relatives on admission, some of them had to endure hardships of having to survive harsh weather and poor accommodation while they waited to fulfil their “responsibilities”. An observational tour around major health facilities showed several instances where relatives of patients spent nights and days on virtually bare floors in the open within and around hospitals. Many attributed their situation to long distances to health facilities as well as the lack of financial resources to commute to the health facilities every day.

*…My daughter has just delivered. …There were complications, so she was referred here* [Okomfo Anokye Hospital] *…I have spent four days here now. …I sleep* *at this place* [Bare floor along a pavement]. *…We live far from the hospital….We are from the Western Region* [another administrative region of Ghana] (Domaa 55 years, female).

Such incidents had ramifications for the carers’ health, which in most cases were unanticipated and not factored into their actions. Therefore, one could speculate confidently that the effects of BSC was not felt only among inpateints and health facilities but also the careers themselves whose sacrifices had negative consequences for their health aside from time lost from other productive ventures.

## Discussion

The study has examined the effect of BSC in the course of delivering health care for inpatients. The findings revealed both negative and positive effects of the tight-knit social relationships of inpatients. Despite the negative effects, the findings showed that it would not be entirely inaccurate to stipulate that patients without familial support while on admission are likely to be deficient in quintessential support required to ensure effective and timely recovery considering the inefficiencies in the health system (Saleh, 2013). The contradictory findings nonetheless, help to clarify the dilemma about the role of BSC in care delivery (Reeves et al., 2014). The findings do project the close social ties of inpatients as a complement to care delivery rather than as a substitute for the efforts of the formal system. The presence and efforts of familial networks ensure that prescriptions by the supply side of care continuum are accepted and ultimately adopted due to trusting relationship with their acquaintances (Gilson et al., 2005), as some participants demonstrated by continually confiding in their acquaintances while on admission.

According to the findings, one can speculate that there was some degree of distrust and unhealthy relationship between the public and the health system. This explains why relatives of the sick persons felt uncomfortable with the idea of letting health professionals entirely care for the sick and why patients rather discussed their new symptoms to relatives rather than health professionals. The observation also alludes to the very nature of BSC as consisting of strong social ties as opposed to the relationship between health personnel/system and the public, which may be labelled as a form of bridging or even linking social capital (Grootaert & van Bastelaer, 2002; Halpern, 2005). Furthermore, this mistrust, to a significant extent, explains some of the challenges of Ghana’s health system such as late recourse to medical help among the public which is known to result in poor health outcomes especially among rural populations (Baiden et al., 2006). However, others opined that the presence of relatives tends to make patients less forthcoming about how they feel due to predictably shyness and fear of disappointing their acquaintances by disclosing discouraging information (Mazer et al., 2014). Therefore, the happenings cannot be attributed entirely to poor relationships with health personnel. Nonetheless, Østergaard (2015) acknowledged from a review of related literature that trust between patients and health providers is a key ingredient towards improving the health sector in sub-Saharan Africa. Low levels of trust apparently explain the insistence of networks of patients on making themselves known and available to health professionals while their relatives received care. The presence of relatives and patients are known to encourage health professionals to give their best, even in developed countries (Alhassan et al., 2015; Labrecque et al., 1991). The behaviour of the close relatives is, therefore, consistent with those of others in other contexts although the degree of intrusion in care delivery may differ (Labrecque et al., 1991). Moreover, this viewpoint held by the participants exudes the poor monitoring and supervision of personnel in the health system (GHS, 2015; Saleh, 2013). The fact that relatives of inpatients felt the need to hang around hospitals and monitor proceedings by themselves support this assertion. Indeed, studies about Ghana’s health system advocate for “in-service training programmes coupled with supportive supervision programmes [to] help ensure that guidelines are followed and standards maintained” (Saleh, 2013, p. 42).

Moreover, the positive response to decisions of relatives even against medical advice raises issues about cultural sensitivity of health systems—something which is missing in the practice of many facilities and systems across sub-Sahara Africa (MacIntyre et al., 2013). According to the findings, this can be attributed to the push by close ties of inpatients—who may sometimes not be in the position to adequately make their own decisions. Many individuals, households and even communities in Ghana tend to grapple with a multiplicity of medical modalities in the light of cultural and belief complexities (Gyasi et al., 2016a; 2016b). Apparently, the critical role of BSC explains the call for a move away from individualistic focus in healthcare delivery to a more holistic approach—one that considers the broader social being of a person by incorporating their social ties in healthcare delivery (Levine & Zuckerman, 1999; Lewis et al., 2014). Nonetheless, it is equally vital to note that some relatives—as previous studies warn—tend to conflate or present their personal beliefs and values as that of inpatients (De Allegri et al., 2015; Mazer et al., 2014). They often do that by making healthcare decisions without recourse to the choice of the person in need when they have the opportunity to contribute and thereby potentially endangering the health of the sick (De Allegri et al., 2015; Mazer et al., 2014). Notwithstanding, studies indicate that people with chronic and severe health conditions for instance, actively rely on their family and friends for information and emotional support to manage their conditions and for companionship (Gilbar, 2011; Korfage et al., 2013; Levine & Zuckerman, 1999; Roddis et al., 2016). Furthermore, by engaging actively with the close ties of inpatients, the health facilities are bound to become more culturally sensitive in line with the vision of the World Health Organization (WHO, 2013). Considering these assertions, together with the contribution of close ties of sick persons as observed here make decision about involvement of BSC in care delivery a complicated and yet a necessary one.

Despite the physical disruption or interference in healthcare delivery, elimination of such incidents (interfering with care) was extremely difficult considering that many health facilities could not do without the resources embedded in inpatients’ tight-knit relationships including financial support, provision of food, and laundry services. It was quite surprising to observe that financial support to inpatients by their relatives was a common phenomenon. This is in view of the fact, that there is a social health insurance policy in place in Ghana—the National Health Insurance Scheme (NHIS) (Schieber, Cashin, Saleh, & Lavado, 2012). However, a recent evaluation of the policy shows a rather dismal outlook as out-of-pocket payments have soared as a result of several socio-economic and administrative challenges (Gros, 2016; Saleh, 2013; Schieber et al., 2012). The weak state of the NHIS apparently explains why the inpatients needed to be supported financially by their close acquaintances. In the context of this study, this partly explains why the relatives were allowed to spend nights within and around hospital premises as they waited to assist health personnel and their relatives. Moreover, reliance on one’s social acquaintances for financial support for health care purposes is common in the study context (Amoah & Phillips, 2017; Moyer et al., 2014). Indeed, scholars argue that people in societies with weak social security systems may have to depend on their social networks for different kinds of material and economic support (Rostila, 2013).

Furthermore, availability of such relatives, despite their disruptive behaviour sometimes, offers an opportunity for health personnel to learn more about the health conditions of patients and the potential impact of the illness on not only the patient but also his/her entire social networks (Creasy, Lutz, Young, & Stacciarini, 2015). Such knowledge promotes proper healing and well-being. It also expands perspective on the best treatment options by situating the entire process within the broader social structure of the patient including their social norms and practices concerning health as acknowledged by the seminal work of Berkman (1995). There is moreover systematic evidence across the globe as regards agitations between health personnel and visitors in general hospitals (Çelik, Çelik, Ağırbaş, & Uğurluoğlu, 2007; Hahn et al., 2008; Levine & Zuckerman, 1999). In places such as Turkey, physical and verbal abuse of health personnel by relatives of patients have been recorded (Çelik et al., 2007). The situation in this study is thus, not peculiar to the study context (Hahn et al., 2008). In Ghana and many other contexts, one can attribute such physical confrontations to lax security at various hospitals to help prevent intrusions into health facilities. Hospital administrators and the health system at large must, in the view of this, take precautions to secure the safety of the sick and health personnel.

Although some of the health professionals attributed the poor behaviour of close ties of inpatients to low health-related knowledge, the happenings in the respective facilities—including the need for relatives to provide assistance to care delivery—rather exposes the gaps in the system as others have also observed in Ghana (Baneseh, 2016; Saleh, 2013). The low knowledge levels as claimed by some health personnel partly manifest the undefined role for social networks of inpatients. This knowledge gap leaves relatives and acquaintances of patients wandering. In their desperate attempts to gain information and to contribute to caregiving, they end up interfering in the healthcare process. Such desperate attempts encourage relatives to spend nights in unsavoury conditions even to the detriment of their health. These behaviours are, however, explained by Levine and Zuckerman (1999, p. 148) who noted that “family caregivers want understandable and timely information, better training, compassionate recognition of their anxiety, guidance in defining their roles and responsibilities, and support for the setting of fair limits on their sacrifices”. Therefore, to involve relatives in the course of healthcare delivery, a clear role may have to be defined aptly for effective and continuous support (Makoae & Jubber, 2008). This position is also in tandem with the notion of Whittaker and Van Beveren (2005) who see social capital as an avenue for fluid sharing of information and knowledge between individuals and groups who share a goodwill—healing the sick in this case, despite the tremendous challenges in leveraging it. However, this proposal can only come to fruition once the entire health system and even health facilities acknowledge one thing: that “health promotion rests on the shoulders of not only individuals but also of their families and communities” which will pave way to “commit resources over the next decade to designing, testing and implementing interventions in this area” (Berkman, 1995, p. 245).

While this paper has aptly elucidated the contribution and influence of close ties of inpatients on care delivery, it must be stated that the observations and subsequent analyses are devoid of the views of that of patients themselves. Further studies could extend this work by ascertaining the views of patients regarding how they juggle between the information and support they receive from health professionals and their social network. The findings of such studies could help to unravel further intricate details of how BSC shapes healthcare delivery for inpatients. Notwithstanding, the findings provide a fruitful starting point for addressing this gap in healthcare delivery in Ghana and other contexts. The study also used a small sample of primary participants, health personnel, and health facilities. The sample size may have compromised the depth of the data. However, the data collection process was guided by the concept of theoretical saturation—the point at which no new knowledge emerged from the interviews (Bryman, 2012).

## Conclusion

We examined the role of BSC of inpatients in healthcare delivery in Ghana. The findings showed that considering the weaknesses in the local health system, the contribution of relatives and friends of inpatients are vital for efficient healthcare delivery. Nonetheless, the tendency of relatives to interfere with the process and the potential dangers of such behaviour to their health raised a cause for concern. Given these, it is suggested that the BSC should be engaged in a manner that enhances its benefits for healthcare delivery. It will be prudent to explicitly define a role for at least the immediate social acquaintances of inpatients who can be identified at the point of entry into health facilities. The role should be carved in a manner that does not infringe on the privacy of the patients; instead grants relatives the opportunity to aid in healing through informational, instrumental, or emotional terms. Also, information on health conditions of inpatients and treatment regimen—with permission from patients, should be fed to the identified social relations of the sick in a bid to inhibit the adverse influence of BSC on healthcare delivery.
